# A rare case of incarcerated obturator hernia in an elderly female: Importance of timely diagnosis and choice of surgical intervention – open or laparoscopic

**DOI:** 10.12669/pjms.40.11.9994

**Published:** 2024-12

**Authors:** Vladimir Resanovic, Aleksandar Resanovic, Zlatibor Loncar

**Affiliations:** 1 Vladimir Resanovic, MD, PhD, Assist Professor of Surgery, Medical Faculty, University of Belgrade; Clinic for Emergency Surgery, Emergency Center, University Clinical Center of Serbia, Belgrade, Serbia; 2 Aleksandar Resanovic, MD, PhD, General Surgeon, Dubai London Hospital, Dubai, UAE; 3 Zlatibor Loncar, MD, PhD, Associate Professor of Surgery, Medical Faculty, University of Belgrade; Clinic for Emergency Surgery, Emergency Center, University Clinical Center of Serbia, Belgrade, Serbia

**Keywords:** Obturator, Hernia, Incarceration, Intestinal, obstruction

## Abstract

Obturator hernia is a rare clinical condition, mainly affecting the elderly and frail patients. It is often challenging to diagnose, and carries a significant morbidity and mortality risk. We aim to highlight the importance of timely and adequate diagnosis followed by early intervention that can be done as open or laparoscopic. Furthermore, through a literature review, we intend to compare two types of surgical approaches (open vs. laparoscopic). Herein, we present the case of an 83 years old female admitted as an emergency with right lower quadrant, colicky abdominal pain, followed with nausea, vomiting and constipation. Computed Tomography (CT) scan revealed a right-sided incarcerated obturator hernia with proximal bowel distension. Emergency surgery was performed, emphasizing the importance of early intervention to prevent bowel necrosis and the need for bowel resection; due to asthma we opted for open approach. Intraoperatively, a right-sided incarcerated obturator hernia was confirmed, with a segment of small bowel herniating through the obturator canal.

We discuss the role of CT scanning in diagnosis, the necessity of prompt surgical management, and the possibility of open and laparoscopic approaches through a literature review. The choice between open and laparoscopic approaches should be individualized, considering the patient’s clinical status and the potential risks and benefits of each technique. Further research comparing the long-term results of open versus laparoscopic repair in incarcerated obturator hernia is something to be explored in the efforts of achieving the highest standards of patient care and maximizing the chances for an optimal treatment outcome.

## INTRODUCTION

With an incidence rate of 0.07-1% of all hernias,^1^ obturator hernias are a rare, yet significant clinical finding. They occur when abdominal contents protrude through the obturator foramen, leading to incarceration and potential bowel obstruction.^2^ Due to their nonspecific clinical presentation and their localization being deep within the pelvis, obturator hernias are particularly challenging to diagnose.^3^ Prompt diagnosis and surgical intervention (either open or laparoscopic) are of utmost importance in preventing bowel necrosis and associated complications.^2^,^4^ The population at risk are frail, elderly women that are often malnourished.^2^ CT imaging plays a crucial role in establishing diagnosis.^2^ Here, we present a case highlighting the significance of early recognition and management of an incarcerated obturator hernia (IOH) in an elderly female patient.

## CASE PRESENTATION

**Fig.1:**
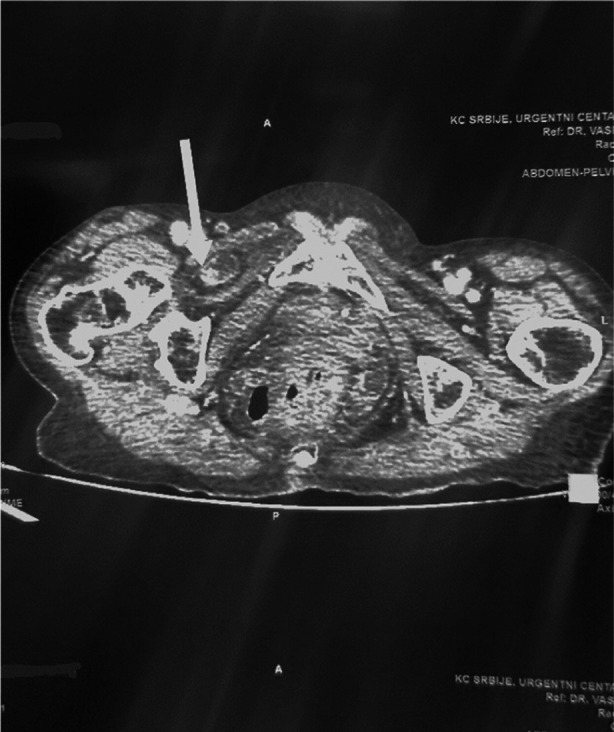
Incarcerated small bowel on CT (arrow).

An 83 years old female presented to the emergency department with acute-onset of right lower quadrant, colicky abdominal pain, followed by intermittent nausea, vomiting and constipation. On examination, the patient appeared frail and malnourished, with impaired pulmonary function due to asthma. Abdominal examination revealed tenderness in the lower quadrants with no palpable masses. Bowel sounds were *hyperactive*. Given the clinical suspicion of bowel obstruction, backed up with an abdominal ultrasound that visualized a distended small bowel loop in the pelvis, we opted for a CT scan. A CT scan of the abdomen and pelvis was promptly performed; the findings revealed a right-sided IOH with a proximal loop of the small bowel measuring up to 28mm in diameter. Distal loops of the small bowel appeared collapsed, suggestive of bowel obstruction secondary to herniation (Fig.1).

### Management:

**Fig.2:**
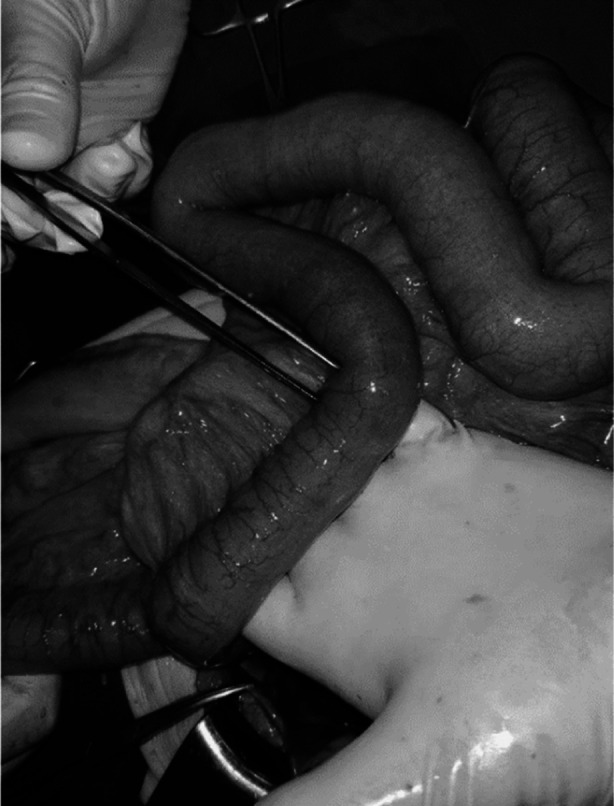
The herniated bowel without the signs of ischemia.

In light of the CT findings and the patient’s clinical condition, an indication for an emergency surgery was established. Informed consent for operative treatment was obtained from the patient. The patient was taken to the operating room for emergency laparotomy. Due to anesthesia-related issues (asthma) we opted for an open approach. Intraoperatively, a right-sided IOH was confirmed, with a segment of small bowel herniating through the obturator canal. Deliberation of the small bowel was performed, followed by a herniorrhaphy. The herniated bowel appeared edematous but viable (Fig.2). The surgery proceeded uneventfully, and the patient tolerated the procedure well.

### Outcome:

Postoperatively, the patient was monitored closely in the surgical intensive care unit and then in the surgical ward from the second postoperative day. An abdominal drain placed into the Douglas pouch and a nasogastric tube, were removed on the second postoperative day. Her recovery was uneventful, with the resolution of abdominal pain and the return of bowel function. Oral intake was gradually resumed on the second postoperative day, and the patient’s nutritional status was optimized during the hospital stay. She was discharged home in stable condition with appropriate follow-up arrangements. The patient was fine after surgery on a six-month follow-up.

## DISCUSSION

IOH, though a rare occurrence, due to its high morbidity and mortality rates, especially among the elderly population, remains a challenging condition in the medical field.^2^ The obturator canal continues to the obturator foramen, through which the obturator nerve and associated blood vessels pass. It is up to 3-cm long and 1-cm wide and is filled with fatty tissue. The loss of adipose tissue in lean individuals makes the obturator canal permeable to intra-abdominal contents, most often the small intestine.^3^

An increase in intra-abdominal pressure (which our patient did not have) is also a predisposing factor, and these two factors, when combined, initiate the development of an obturator hernia.^4^ The condition is characterized by the protrusion of abdominal contents through the obturator foramen, creating a deep-seated and often elusive presentation within the pelvis. Diagnosing IOH is challenging due to its complexity and unspecific symptoms related to intestinal obstruction, which are not limited to this condition.

Since the majority of the patients are elderly, and that there is high probability of incarcerated bowel, this underscores the critical need for rapid diagnosis and intervention to prevent severe complications, such as bowel ischemia, necrosis, and potentially, perforation.

The non-specific nature, especially in the early stages, of its clinical presentation often results in the delay of establishing a diagnosis. However, advancements in diagnostic imaging, notably CT, have allowed for a drastically reduced timeframe for diagnosing obturator hernias. In our case report, CT imaging was crucial not only for the confirmation of the diagnosis, but also for creating a path for the subsequent surgical management. The importance of the use of CT imaging in these scenarios cannot be emphasized enough, as it facilitates the early detection and diagnosis that is vital for successful treatment outcomes.

The timing of surgical intervention in cases of obturator hernia cannot be overstressed.^5^ In light of the undoubtable risks for bowel incarceration and strangulation within the obturator foramen, it is a logical clinical premise that any delay in surgical intervention can lead to bowel necrosis and perforation, necessitating bowel resection and significantly altering the patient’s recovery. Early surgical intervention is of utmost importance in preserving bowel viability and minimizing the overall risk of morbidity and mortality. Leeds et al^6^ in their paper showed that delayed surgery was linked to increased rates of complications, prolonged time of operation, longer hospital stay, higher rates of readmission and reoperation, as well as an increased 30-day mortality rate. In our case report, timely surgical management enabled the preservation of the affected bowel, eliminating the need for resection.

Bearing in mind the magnificent achievements that have been made in the field of laparoscopic and robotic surgery, we are cautiously raising a question regarding the choice of surgical approach in the treatment of IOH. Both open and laparoscopic techniques have been documented, each with its distinct advantages and considerations. Laparoscopy, renowned for its minimally invasive approach, offers benefits such as reduced postoperative pain and shorter hospital stays. Nonetheless, concerns regarding the distended bowel loops manipulation with graspers and the potential risk of injury, especially in cases where there is a valid clinical premise of bowel ischemia existence, often inclines the surgeon to opt for the open surgical approach in emergency situations. In an emergency setting, a laparoscopic transabdominal approach facilitates bowel visualization, reduction and resection (if necessary) compared to totally extraperitoneal.^7^ De Figueiredo et al.^8^ in their paper did the analysis of 53 studies in the Pubmed database that addressed to incarcerated obturator hernia.

A total of 425 patients were enrolled in his research, with the mean age of 83.3 years; 419 were female (98.6%) and 6 were males (1.4%). Out of this number, 239 patients underwent open surgery (56.2%) with primary repair; MESH repair has been done in 121 patients (28.5%) using open approach. In the group of patients that underwent laparoscopic repair, 44 (10.4%) had MESH repair and 21 (4.9%) underwent primary repair. Small bowel resection was performed in 171 patients (40%) in the open group and in 10 patients in the laparoscopic group. This study showed that laparoscopic approach is better compared to open approach in the terms of better recovery and recurrence rate. However, the majority of obturator hernias today (85.71% of patients in this study) are still operated on using an open approach without MESH. According to this paper, laparoscopic approach with MESH should be applied in all situations when patient and local conditions allow; further studies are needed in order to determine the best approach in the case of incarcerations and strangulation.

While laparoscopic repair holds considerable benefits under normal circumstances, the necessity of ensuring a safe and effective resolution to an IOH, particularly in elderly patients with potentially compromised vascular supply, underlines the necessity of a careful evaluation of the risks involved. The edema and impaired blood flow to the bowel tissue further complicate the decision-making process, emphasizing the need for immediate and decisive action. The open surgical approach preference in our case report was driven by anesthesia-related issue (asthma).

The management of an IOH, especially in elderly patients, outlines the importance of fast-tracking the diagnostics that guide the path for the surgical intervention. IOH should always be considered as a differential diagnosis in frail elderly women, even in the case of intermittent, paroxysmal pain followed by nausea, vomiting and constipation.^9^ The availability of CT imaging has significantly enhanced our ability to diagnose this evasive condition promptly and plays a crucial role in diagnosis of IOH.^10^ A prompt surgical intervention, via an open or laparoscopic approach, due to the urgency and complexity of these cases, remains vitally important to minimizing complications and improving patient outcomes. Further research comparing the long-term results of open versus laparoscopic repair in IOH is something to be explored in the efforts of achieving the highest standards of patient care and maximizing the chances for an optimal treatment outcome.

## CONCLUSION

Incarcerated Obturator Hernia (IOH) is an extremely rare condition, affecting mainly the elderly population. CT imaging plays a crucial role in confirming the diagnosis and guiding surgical management. Timely surgical exploration facilitates the preservation of bowel viability and minimizes the risk of complications. The choice between open and laparoscopic approaches should be individualized, considering the patient’s comorbidities and the potential risks and benefits of each technique.

### Authors Contribution:

**VR:** Drafting the article, substantial contribution to analysis, acquisition of data, responsible for the accuracy and integrity of the work.

**AR:** Contribution to conception, drafting the article and Review

**ZL:** Critical revising of the article, final approval of the version to be published.
